# Bioinformatics in the Netherlands: the value of a nationwide community

**DOI:** 10.1093/bib/bbx087

**Published:** 2017-09-15

**Authors:** Celia W G van Gelder, Rob W W Hooft, Merlijn N van Rijswijk, Linda van den Berg, Ruben G Kok, Marcel Reinders, Barend Mons, Jaap Heringa

**Affiliations:** 1 Dutch Techcentre for Life Sciences (DTL), Utrecht, Netherlands; 2 Delft University of Technology, Delft Bioinformatics Lab, Dept. of Intelligent Systems, Faculty of Electrical Engineering, Mathematics and Computer Science, Mekelweg Delft, Netherlands; 3 Vrije Universiteit Amsterdam, Amsterdam, Noord-Holland, Netherlands

**Keywords:** bioinformatics community, bioinformatics training, interoperability, fair data principles, national research infrastructure, bioinformatics research

## Abstract

This review provides a historical overview of the inception and development of bioinformatics research in the Netherlands. Rooted in theoretical biology by foundational figures such as Paulien Hogeweg (at Utrecht University since the 1970s), the developments leading to organizational structures supporting a relatively large Dutch bioinformatics community will be reviewed. We will show that the most valuable resource that we have built over these years is the close-knit national expert community that is well engaged in basic and translational life science research programmes. The Dutch bioinformatics community is accustomed to facing the ever-changing landscape of data challenges and working towards solutions together. In addition, this community is the stable factor on the road towards sustainability, especially in times where existing funding models are challenged and change rapidly.

## Introduction

The term ‘bioinformatics’ was coined by Paulien Hogeweg of Utrecht University (the Netherlands) as early as 1970. She defined it as ‘the study of informatic processes in biotic systems’ [1]. Hogeweg recognized that information processing is one of the defining properties of life, taking place at multiple levels and across different timescales. For instance, DNA information processing underlies various intracellular and intercellular processes during a lifetime, but information also builds up during evolution. Basically, the vast numbers of molecules in an organism constitute a flexible information processing system that can interact with its environment. Hogeweg realized that information processing could serve as a useful metaphor for understanding living systems. Therefore, she concluded that it was useful to distinguish bioinformatics as a research field in addition to biophysics and biochemistry. (Note that she initially called it a ‘working concept’ rather than a research field.) Following these early developments, the Netherlands has continuously played an active role in the field. For example, in the 1980s, seminal groups at the University of Groningen developed multiple internationally renowned techniques for biophysics, molecular biology and bioinformatics. Principal investigators included Herman Berendsen, Wilfred van Gunsteren, Jan Drenth and Wim Hol. All of these developments have acted as a breeding ground for later national initiatives, such as the Netherlands Bioinformatics Centre (NBIC), the first pan-Dutch bioinformatics initiative.

In this article, we describe the process of building the bioinformatics community in the Netherlands since 2003, under different working and funding models. We also discuss the current situation and conclude with our future plans, including acquisition via the Dutch Roadmap Large-Scale Research Infrastructure, where the FAIR principles [2] play a crucial role.

## NBIC (2003–13)

### Bioinformatics under the Netherlands Genomics Initiative

**Figure 1:**
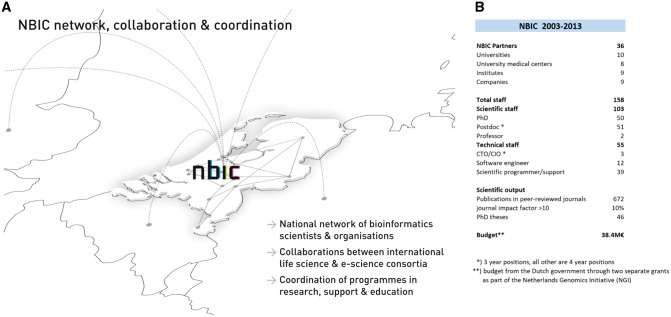
**(A** and **B)** Key facts and figures about NBIC, for the period 2003–13.

In 2002, the Netherlands Organization for Scientific Research (NWO, the main governmental science funding organization in the Netherlands) started the Netherlands Genomics Initiative (NGI). Its purpose was to advance genomics research in the Netherlands and to valorize the results. NBIC, established in 2003, was one of the funded organizations; it received over EUR 38M in the period 2003–13. Seminal scientists at the cradle of NBIC were Gert Vriend, Bob Hertzberger and Jacob de Vlieg. NBIC’s mission was to create a strong bioinformatics community that performs internationally competitive bioinformatics research and can cope with the demand for bioinformatics expertise, infrastructure and personnel in the Dutch agro, food and health sectors. In the decade of its existence, NBIC became the national bioinformatics hub, connecting bioinformatics research groups at the level of PIs (the NBIC faculty), young scientists, programmers and teachers in the Netherlands. Activities were closely entangled with those of the Dutch life science and e-science initiatives, especially in the NGI programmes. The NBIC consortium consisted of nearly all Dutch universities, university medical centres, research institutes and an initial few industrial partners. We used long-term agreements to build an open and sustainable partnership of organizations keen to develop and apply bioinformatics. A lightweight legal framework was developed for partner and project contracts to facilitate the inclusion of new NBIC partners and to allow for straightforward intellectual property protection. Figure 1 provides an overview of key NBIC facts and figures.

During the NBIC period, we continuously aligned our activities with the international field. In addition, an International Advisory Committee oversaw and safeguarded NBIC’s positioning in the global bioinformatics landscape. During an external review in 2011, an invited international review committee described NBIC’s overall accomplishments as ‘impressive’. The committee indicated that a coordinated effort in a national centre like NBIC appeared to be the best way to build a computational biology and bioinformatics faculty. Furthermore, the committee welcomed the integrated model of research, support and education, which were organized in the three NBIC programmes BioRange, BioAssist and BioWise, respectively.

### Research: BioRange

In NBIC’s research programme BioRange, 101 PhD and postdoc positions were distributed over bioinformatics groups at universities and university medical centres in the Netherlands (see also Figure 1). BioRange research was a mix of ‘applied research’ driven by biological questions raised by partner centres (mostly NGI-funded), and technology/methodology driven research initiated by the bioinformatics groups themselves. The research programme was focused on four topics: sequence-based bioinformatics, genotype–phenotype modelling, proteomics and metabolomics and systems bioinformatics. Elements that were evaluated positively by the international review committee were the scientific productivity, the balance between fundamental and applied bioinformatics research and the integration of the BioRange and BioAssist programmes (see below), which enabled research products to be developed into applications for life scientists.

### Support: BioAssist

The BioAssist programme focused on bioinformatics support. Its tagline was ‘making other people’s data work’. In the first 5 years of NBIC, experiences with a central software service had been mixed. Problems were mainly attributed to the lack of expert assistance for end users of bioinformatics software. To strengthen the support expertise capacity underpinning resources, a service team was started as part of BioAssist. The team helped researchers throughout the country with small projects by locally stationing a support team member for a short period of time (typically 1 month). Furthermore, 20–25 scientific programmers were permanently stationed at the bioinformatics expertise groups of NBIC’s partners. These programmers collaborated in six large thematic projects: Next-Generation Sequencing, Proteomics, Metabolomics, Functional Genomics, Systems Bioinformatics and Biobanking. The projects were managed by a group of central technical coordinators that were part of the NBIC core team. They dedicated half of their time to the coordination of the six thematic projects. The other half of their time was spent on collective (re-)engineering of selected output from the NBIC research groups into more stable and performant software tools suitable for use by others (from ‘professor ware’ to professional ware). NBIC supported a collective Trac project management and bug tracking environment for each project involving BioAssist software engineers. Many of these projects have since migrated to GitHub. Tools from NBIC can be found in the ELIXIR bio.tools repository under the flag ‘ELIXIR-NL’. BioAssist has been instrumental in strengthening the interaction with many basic and translational life science research initiatives in the Netherlands, such as in rare diseases, crop biology, industrial microbiology, translational medicine (CTMM-TraIT initiative) and clinical genomics (e.g. Genome of the Netherlands project in BBMRI-NL). Further, the BioAssist activities led to the key involvement of the bioinformatics field in establishing the Dutch computational science grid infrastructure between 2007 and 2012 (BigGrid), not further described here. The international review committee regarded the BioAssist model to be a crucial element for the success of the NBIC model and advised to continue this model and make it sustainable.

### Education: BioWise

Education has been an essential element of the NBIC strategy from the start. The mission of NBIC’s education programme BioWise was to train the current and next generation of bioinformaticians and biologists. BioWise target audiences ranged from the general public [3], and high school pupils, to PhD students and more senior researchers.

With strong commitment of a large group of bioinformatics PIs, BioWise has realized a diverse course programme for PhD students and other researchers in bioinformatics and other life science disciplines. This resulted in the set-up of the NBIC PhD School, which has been up and running since 2009. The PhD course programme was structured in a Technology Track, an Application Track, and a Life Sciences Track. Important elements in the set-up of the courses were the fixed 1-week format of Technology and Application Track courses, and the fact that these courses were developed by PIs from at least two different institutions. An important side effect of the latter was the strengthening of the bioinformatics community. In 2014 (after NBIC ended), the BioWise course programme was incorporated in the curriculum of the Netherlands Bioinformatics and Systems Biology Research School (BioSB, see further below).

Another important community development was the foundation of the Regional Student Group (RSG) Netherlands in 2008. This is a group of bioinformatics PhD students in the Netherlands, which is part of the worldwide network of RSGs coordinated by the Student Council of the International Society for Computational Biology (ISCB). It is active in organizing national activities for PhD candidates [4, 5], such as company visits, BioCafes and PhD Retreats. In addition, it has been involved in co-organizing several editions of the International Student Council Symposium during ISCB’s annual ‘Intelligent Systems in Molecular Biology’ (ISMB) conference.

BioWise has continuously reached out to international organizations, making sure to share expertise and avoid reinventing the wheel on training and training organization. It has been active in international organizations such as the Bioinformatics Training Network [6, 7] and the Global Organisation for Bioinformatics Learning, Education and Training (GOBLET) [8, 9]. NBIC was one of the co-founders of GOBLET in 2012, and BioSB (see below) is part of its executive board at present.

An example of the BioWise outreach activities is the high school project Bioinformatics@school (www.bioinformaticsatschool.eu, www.bioinformaticaindeklas.nl). This project started in 2006 as part of the national programme called Traveling DNA labs (www.dnalabs.eu, www.dnalabs.nl), where teaching assistants physically visit high schools throughout the country. They turn the classroom into a laboratory and bring modern life science and bioinformatics research alive for high school students and teachers. To date, >23 000 high school pupils have attended a Bioinformatics@school practical at their own school.

The international review committee judged the BioWise output to be ‘phenomenal’ and commended the fact that the full spectrum of target audiences was being served. The review committee appreciated that all this was accomplished with minimal funding and mainly based on in-kind contributions of the Dutch bioinformatics PIs, demonstrating the significance of the network that had been built.

## Reorganizing the Dutch bioinformatics landscape without central funding (2014-now)

### Materialization of DTL, ELIXIR-Netherlands and BioSB

In 2012, it became clear that the research funding climate in the Netherlands would substantially change: funding of large, public–private research and innovation programmes would not be sustained by the Dutch government. For the Dutch bioinformatics field, this instigated a change in its mode of organization, operation and funding. NBIC joined forces with three other technology centres that were previously funded by NGI (Netherlands Proteomics Centre, Netherlands Metabolomics Centre and Netherlands Consortium for Systems Biology, NCSB). They jointly prepared a new initiative that would operate as an expertise network, building on the results obtained, technologies developed and facilities set up in the period 2003–13. This new initiative was named the Dutch Techcentre for Life Sciences (DTL, https://www.dtls.nl/). From its formal start in 2013, DTL has had a strong focus on organizing data stewardship in the Dutch life sciences and working towards a nation-wide bioinformatics infrastructure. In addition, DTL became the Dutch node of the European Life-Science Research Infrastructure ELIXIR (ELIXIR-NL, https://www.dtls.nl/elixir-nl/) in 2014, forming the bridge to bioinformatics groups in other European countries. In parallel, the Netherlands Bioinformatics and Systems Biology Research School (BioSB, www.biosb.nl) was founded in 2014 to safeguard the education activities of BioWise.

### DTL: mission and organization

**Figure 2:**
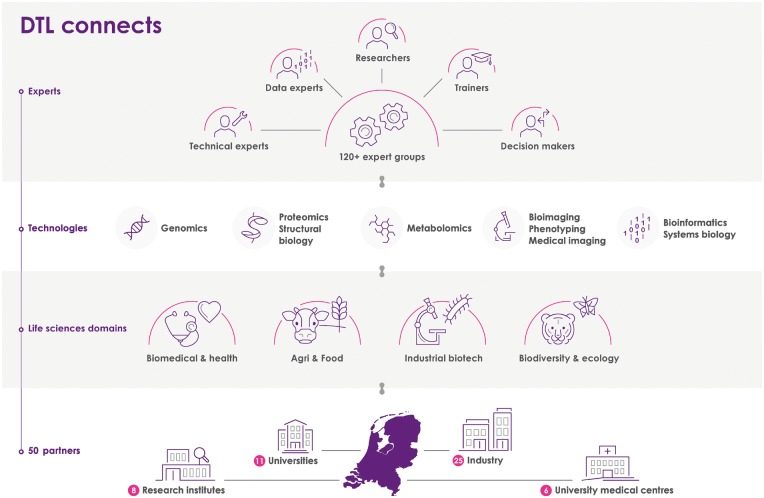
Overview of the set-up and scope of DTL; numbers refer to the situation in June 2017.

DTL’s mission is to establish an interconnected research infrastructure that enables cross-disciplinary and data-intensive life science research in national and international collaboration. DTL’s main themes are accessible high-end technologies, FAIR data treatment and expert training. To be able to function independently from uncertain government funding, DTL was set up as a public–private partnership of academic and commercial providers of technologies. Each partner pays an annual partnership fee. Summer 2017, DTL has grown into a partnership of 50 life science organizations (Figure 2), many of which were also active in one of the NGI-funded centres in the period 2003–13. It encompasses nearly all Dutch universities and university medical centres, and a rapidly growing number of institutes and companies. The DTL partners have a collective interest in realizing a high-quality interconnected research infrastructure, and they actively contribute to the DTL network. Partner organizations are directly involved in the DTL governance through a representative in the partner advisory committee (PAC).

#### Network of expert groups

DTL is first and foremost an expanding network of >120 expert groups (https://www.dtls.nl/technology-hotels/list/; see also Figure 2). Many of these were also active in one of the NGI-funded technology centres in the period 2003–13, and a substantial number are specialized in bioinformatics. DTL offers a platform for researchers, programmers and teachers to exchange, to create solutions for common challenges and to coordinate the development of guidelines and standards. The expert groups are

- specialized in wet-lab technologies ranging from genomics, proteomics, metabolomics and advanced microscopy to medical imaging and high-throughput phenotyping, and in dry-lab technologies ranging from bioinformatics, (medical) informatics, e-science and computational (systems) biology;
- involved in life sciences domains ranging from health to agro, nutrition, industrial microbiology, ecology and biodiversity;
- located at universities, university medical centres, research institutes or private companies across the Netherlands (these are the DTL partners; one DTL partner may host multiple expert groups); and
- providing access to their local facilities for projects outside their group, a construction that we refer to as ‘Technology Hotels’; this concept will be explained later in this article.

#### DTL network organization

DTL is run as a public–private network of experts and policymakers affiliated with the DTL partner organizations, supported by a foundation (‘Stichting DTL’) with a small facilitating team. This team (the ‘DTL core team’) supports and guides the activities of the DTL community. The core team’s activities include (1) showcasing inspiring partner initiatives; (2) facilitating the exchange of expertise; (3) driving active collaboration among DTL-associated groups; (4) organizing topical science and technology meetings; (5) providing information to the community; (6) guiding collective agenda-setting among partners, government and funders; (7) international outreach of important deliverables of the DTL collective; and (8) assisting in the architecture of cross-partner projects and acquisition of funds to execute them.

#### DTL Projects organization

To facilitate the acquisition and management of larger-scale projects, DTL has a legal structure available for collaborative (inter)national projects that are connected and aligned by DTL. DTL inherited the formal components developed during the NBIC years, including a Partner Agreement plus associated IP regulation signed by virtually all Dutch research organizations. DTL now provides an ideal stepping stone for collaborative technology research and infrastructure projects among expert groups of DTL partners.

### ELIXIR-NL: mission and organization

In parallel with the development of the DTL network and in collaboration with bioinformatics groups all over Europe, the Dutch bioinformatics community has been involved in the setup of the European research infrastructure ELIXIR (http://www.elixir-europe.org) as part of the ESFRI programme. ELIXIR’s mission is to manage and safeguard the increasing volume of data generated by life science research. It coordinates and sustains bioinformatics resources across its member states. In addition, it helps researchers to more easily find, share and analyse biological data.

ELIXIR follows a hub-and-nodes model, with a single hub hosted at EMBL-EBI in Hinxton (UK) and a growing number of national nodes located at centres of excellence throughout Europe. At the time of writing, ELIXIR comprises 20 national nodes, with EMBL-EBI constituting a separate node. In January 2014, the ELIXIR Consortium Agreement was signed by the Dutch Minister of Education, Culture and Research, making the Netherlands the seventh country to join the ELIXIR research infrastructure. DTL hosts the Dutch node of ELIXIR (ELIXIR-NL).

ELIXIR’s activities are organized in five overarching platforms: Data, Tools, Interoperability, Compute and Training. ELIXIR-NL is actively involved in the latter three, focusing on (a) standards and tools for data interoperability and exchange, (b) computing and storage services and (c) training and education. At present, ELIXIR-NL is also represented in the leadership teams of the ELIXIR Interoperability and Training Platforms. In addition, ELIXIR has four use cases, namely, Rare diseases; Plants; Human Data; and Marine Metagenomics, and ELIXIR-NL is actively involved in the first three. Furthermore, ELIXIR-NL has initiated the development of two new use cases (Metabolomics and Proteomics) for the ELIXIR work programme 2019–23 in collaboration with other ELIXIR nodes.

### DTL/ELIXIR-NL activities: data, technologies and learning

DTL/ELIXIR-NL has established three programmes:

- DTL Data [10], focusing on facilities, tools, resources and expertise to process, analyse, preserve, share, combine and publish data in a FAIR manner;
- DTL Technologies, focusing on high-end wet-lab facilities and the associated expertise, quality assurance processes, best practices, guidelines and standards; and
- DTL Learning, focusing on training and education of users and experts in the fields of Data and Technologies.

Within these programmes, the DTL-associated professionals interconnect and improve the existing research infrastructure by:

- making it findable and accessible;
- aligning and improving quality’
- building new infrastructure; and
- connecting to infrastructures outside the Netherlands.

We will discuss highlights of these activities below.

#### Technology Hotels network

As explained above, the DTL-associated expert groups have registered their facilities or expertise as public and private Technology Hotels. A Technology Hotel offers high-end technology or data expertise to life scientists from all over the Netherlands, on a collaborative or cost-recovery basis. Guests are life scientists that have no access to these particular technological facilities or expertise at their home institute. They receive technical or data support at the hotel. Examples of specific DTL activities are:

- managing an overview of Technology Hotels and what they have to offer (https://www.dtls.nl/technology-hotels/list/);
- organizing funding to acquaint new hotel guests with the potential of the advanced technologies offered by the Technology Hotels through the ZonMw/NWO ‘Enabling Technologies Hotels programme’;
- exchanging best practices, harmonizing data generation and treatment processes and developing standard operating procedures; and
- exchanging and implementing FAIR data stewardship methods and practices to ensure that research data can be combined and reused.

#### FAIR data projects

Adequate research data stewardship (i.e. the long-term and sustainable care for research data in a way that allows for data reuse in future scientific projects and by others) is a formidable challenge. DTL-associated scientists have played a key role in developing the FAIR data principles [2]. FAIR stands for ‘Findable, Accessible, Interoperable, and Reusable’, and these principles act as an international guideline for proper data stewardship. Many activities within the DTL Data programme revolve around the FAIR principles, with a strong focus on data interoperability (i.e. combining data from different sources). The DTL network actively promotes FAIR data stewardship in the Netherlands, in Europe and globally, for instance by:

- organizing ‘Bring your own data workshops’ (https://www.dtls.nl/fair-data/byod/) [11], where scientists can translate their data sets into the FAIR format;
- organizing ‘FAIR at the source’ meetings on FAIRifying data with technology communities;
- driving the development of technology and other infrastructure to support FAIR data stewardship (e.g. Data FAIRport, http://www.datafairport.org/);
- developing innovative methods to make privacy-sensitive data accessible in a safe way (e.g. the ‘personal health train’ project, https://vimeo.com/143245835);
- engaging with international initiatives that promote data sharing and citation [e.g. the European Open Science Cloud Initiative (https://ec.europa.eu/research/openscience/index.cfm?pg=open-science-cloud) and the Research Data Alliance]; and
- encouraging science funders in the Netherlands and abroad to adopt the FAIR data principles and to incorporate proper data management as a mandatory part of grant proposals.

#### Data-related training and education

**Figure 3:**
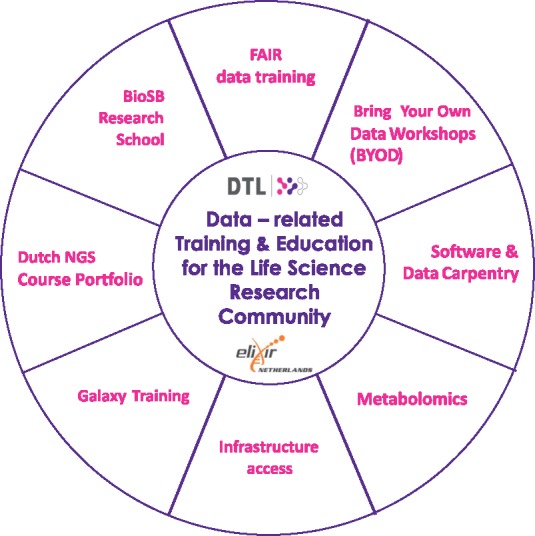
Overview of current themes in the DTL Learning/ELIXIR-NL Training Programme.

Modern life science calls for advanced data and technological expertise. Researchers, programmers and educators need training to acquire such skills. By gathering in a national network, the DTL-associated professionals can identify training needs and develop new courses. This is done in collaboration with the DTL partner institutes, research schools such as BioSB and other initiatives and projects (see below for examples). The efforts of the DTL Learning/ELIXIR-NL Training programme are aligned with and embedded in European (e.g. ELIXIR), and global (GOBLET) training initiatives. Training topics and themes that we are currently working on [12] are summarized in Figure 3 and listed below:

- FAIR data training, Bring Your Own Data Workshops and training in research data management (in collaboration with the DTL Data programme);
- Software and Data Carpentry (SWC/DC, teaching researchers computing skills [13]), in collaboration with NLeSC and SURF, and aligning these with ELIXIR SWC/DC initiatives [14];
- Metabolomics training, in collaboration with the Netherlands Metabolomics Centre, and with EmTraG, the European Metabolomics Training Coordination Group, www.emtrag.eu, which was founded in 2016;
- Infrastructure access (with SURFsara);
- Galaxy training (with the Dutch Galaxy Working Group); and
- Bioinformatics and systems biology (with BioSB) and next-generation sequencing courses (with BioSB and the DTL Next-Generation Sequencing Interest Group).

In addition, we aim to keep an up-to-date inventory of existing education and training in the field of bioinformatics and systems biology, which is automatically transferred to the ELIXIR training portal TeSS (https://tess.elixir-europe.org/).

### BioSB: Netherlands Bioinformatics and Systems Biology Research School

As indicated above, BioSB started its activities in 2014. It was officially launched in 2015 as a follow-up of the NGI-funded research and education initiatives in bioinformatics (NBIC) and systems biology (NCSB). Hence, the national bioinformatics and systems biology communities and their education programmes were merged (http://biosb.nl/education/course-portfolio-2/).

The school’s mission is to offer PhD students and postdocs a stimulating and interactive environment for their scientific development and education in integrative bioinformatics and systems biology. In addition, BioSB is the national platform in which the Dutch community of researchers in bioinformatics and systems biology is organized. To meet its goals, BioSB organizes basic and advanced courses, hot topics meetings and a yearly conference.

Furthermore, when BioSB came into existence, the RSG Netherlands mentioned above broadened its scope and also embraced young researchers in systems biology, and subsequently changed its name into YoungCB (Young Computational Biologists, http://www.youngcb.nl/).

The BioSB research school is organized entirely bottom-up by dedicated PIs and is currently funded by member, course and conference fees. All course teachers are PIs and researchers from the community that contribute to the BioSB portfolio in kind. As mentioned above, BioSB is closely liaising with the cross-disciplinary, integrative and data stewardship activities of the Learning Programme of DTL. In addition, it offers anchor points for the European infrastructures in bioinformatics (ELIXIR) and systems biology (ISBE).

April 2017, an international review committee was invited to review and evaluate the BioSB Research School from an international perspective. The committee was asked to advise and offer guidance on BioSB’s activities with particular regard to its viability, vitality and organization. The executive summary of the committee’s report reads ‘The Review Committee congratulates BioSB for doing a great job in the provision of courses generally, and in community building in bioinformatics and systems biology, in particular, which we recognise isn’t easy. Overall, we consider BioSB to be *a unique programme of national importance and relevance*—indeed, “*a National Treasure*”, of which BioSB members should be proud. The conference is increasingly successful, and young researchers are active and motivated—these developments are particularly impressive, *especially in light of the fact that much of the work is done on a voluntary basis*’.

In addition, the committee provided helpful advice on actions that could be taken to achieve sustainability, e.g., by improving the embedding of BioSB in DTL and ELIXIR-NL. BioSB tries to reach sustainability by adopting a combined course fee and membership model, where members are entitled to reduced fees for BioSB courses and the annual BioSB conference. It has become clear that moderate central funding remains necessary for central support functions, until sufficient members would have been attracted to become self-sustainable.

BioSB also organizes an annual conference for its community. This is a follow-up of 9 yearly NBIC conferences (2006–14) and 6 yearly NSCB symposia (2009–14). The third annual BioSB Conference in April 2017 had well >400 delegates, showing a growing community and thereby the potential for a growing membership.

### Recent developments: the Dutch roadmap to community-driven FAIR science and data stewardship

Over the past 15 years, the Dutch bioinformatics field has balanced core data-driven life science research with efforts to organize the enabling aspects of bioinformatics. Building on a nationwide bioinformatics community, we have worked towards a common vision to develop and embed the bioinformatics in the Dutch life science fields. Moreover, through DTL and the involvement in ELIXIR and the global FAIR data initiative, we have established a vision and strategy to connect infrastructures and support data stewardship based on the FAIR principles as a key enabler of science and innovation.

Adoption in 2016 of the FAIR data approach as key to the European Open Science policy is expected to drive the development and implementation of FAIR technology, not only in bioinformatics and life science research. Anno 2017, Dutch teams are strong drivers to seed the creation of a web of ‘FAIR Implementation Networks’, established by global expert communities in various science fields, including those beyond life sciences (see https://www.dtls.nl/go-fair-european-open-science-cloud/). This grassroots initiative aligns fully with the developments towards the Open Science initiatives by the European Commission and national Dutch policy levels, and with the vision to establish the European Open Science Cloud towards a global Internet of FAIR data and services.

At the end of 2016, ELIXIR-NL was placed on the Dutch National Roadmap for Large-scale Research Infrastructures as one of the research infrastructures of strategic national importance (https://www.dtls.nl/wp-content/uploads/2016/12/Roadmap_UK_2016_2020_lowres.pdf, December 2016). At the same time, all other national life science infrastructure initiatives on the roadmap were encouraged to collaborate in DTL and to coordinate their data approach with ELIXIR-NL. DTL and ELIXIR-NL work towards a generic FAIR-based data infrastructure that provides life scientists with an extensive digital research environment to enable cross-disciplinary life science research. We envisage a type of infrastructure that allows researchers to flexibly combine data sets, bioinformatics tools, computational models and ICT platforms. The infrastructure should operate as a virtual workspace to collaborate and execute data-intensive analytics. It will be established and maintained by a broad range of public and private Dutch research and technology organizations and will form the data ‘backbone’ of topical life sciences infrastructures. This model underlies the nascent national infrastructure for personalised medicine and health research (Health-RI) (https://www.health-ri.org/).

Sustainability of the infrastructure beyond the core roadmap subsidy will be based on a broad partnership of ‘users’ and ‘providers’ of data, tools and compute platforms. A pay-for-use model of selected services within the infrastructure has been developed, which will also sustain the core backbone on which the services run. It is anticipated that modest funding will remain necessary to sustain central activities (e.g. helpdesk; training; freemium).

## Looking back: challenges and lessons learned

In this section, we would like to discuss some of the challenges that we have faced since 2003, when NBIC came into existence. One of the challenges during NBIC’s earlier years was the organization’s double-hatted role of building a close-knit national bioinformatics community as a grassroots activity, while distributing project funds within the framework of approved programmes, and hence acting as a ‘top-down’ science funder at the same time. Although the funding has been crucial in bootstrapping and sustaining a large number of bioinformatics projects, this double role did render it difficult at first to foster trust among all groups in the community.

As integral part of the Dutch strategy to build a strong multi-omics research field through NGI, NBIC was expected to enable the research pipelines of the other centres. It has been a challenge to combine this enabling role with the desire to establish a future-proof bioinformatics community. Initially, this led to suboptimal usage of some of the funding from a bioinformatics perspective. In the decade of NBIC and subsequent DTL era, we have developed a strongly networked community that effectively balances bioinformatics research with support and education.

Attracting industry to NBIC turned out to be difficult during the early years. A possible reason for this might have been our focus on building a comprehensive expert community rather than tangible products such as tools and databases. Meanwhile, close to 40% of the PhDs and postdocs trained in the NBIC years have been recruited by life science and technology companies. In addition, the active climate of stimulating pre-competitive public–private research has led to an increased involvement of industry in DTL and BioSB recent years. In fact, the number of industrial DTL partners equals the number of academic DTL partners at the time of writing.

A more recent challenge is the separation of the three NBIC programmes BioRange, BioWise and BioAssist over DTL and the BioSB research school in 2013/2014. This has led to increased efforts and overhead to align the various bioinformatics activities. Complicating factors were the deflated funding environment after 2013, and the fact that the BioSB research school merges bioinformatics and systems biology.

Finally, we are critically dependent on the goodwill of the community for filling the BioSB course programme. Although the community’s commitment is a great asset, it also puts some strain on organizing courses and keeping a tight course schedule, as there is no other specific incentive for the teachers and course coordinators other than their ambition to support this community effort.

## Conclusion

After 10 years of active coordination of the bioinformatics community and programmes within NBIC, the Dutch bioinformatics community has now embedded its activities in three new initiatives that are strongly intertwined:

- DTL, the Dutch Techcentre for Life Sciences;
- ELIXIR-NL, the Dutch node of ELIXIR, which is hosted by DTL; and
- BioSB, Netherlands Bioinformatics and Systems Biology Research School.

These three initiatives involve many stakeholders, thereby capitalizing on the broad network established by the NBIC programmes. The overall expertise and collaborative culture within the Dutch research community has been a crucial asset and incentive to form a broad faculty group with a growing sense of identity and drive to share knowledge. The younger generation has been particularly eager to learn and get connected in courses and in regular scientific or programmers’ meetings.

DTL and ELIXIR-NL now provide the fertile ground for a large palette of scientific and infrastructural projects, with strong involvement of the Dutch scientific community. Topics include (meta)genomics, metabolomics, proteomics, systems biology, personalized medicine and health, FAIR data and FAIR-compliant tooling and computing. The flexible way of organizing the field and the active support of the established network offer a good basis to build an inclusive ecosystem for complex cross-disciplinary life science projects. Except for individual tools, databases and technologies, we have focussed on building a broad expertise community. DTL/ELIXIR-NL is now set to build a tangible broad community infrastructure through which we expect to be able to better showcase international-grade products that come from Dutch bioinformatics and life science work.

## Glossary of terms

- BioAssist: NBIC’s support programme
- BioRange: NBIC’s research programme
- BioSB: Netherlands Bioinformatics and Systems Biology Research School (http://biosb.nl/), which aims to offer a vibrant environment for the scientific development of, and education in integrative bioinformatics and systems biology to PhD students and academic researchers
- BioWise: NBIC’s education programme
- Bioinformatics Training Network (BTN): a community-led project that aims to provide platform supported, pragmatic solutions for the exchange of expertise, training materials and training experiences ([6], http://www.biotnet.org/)
- Bring Your Own Data workshops: workshops where experts assist researchers in making their research data FAIR
- DTL: Dutch Techcentre for Life Sciences (https://www.dtls.nl/), a growing public–private partnership of Dutch life science organizations
- EMBL-EBI: The European Bioinformatics Institute (http://www.ebi.ac.uk/), which is part of the European Molecular Biology Laboratory (EMBL)
- ELIXIR: European Life-Science Infrastructure for Biological Information (https://www.elixir-europe.org/), an intergovernmental organization that brings together life science resources from across Europe
- ELIXIR-NL: The Dutch node of the European life science infrastructure ELIXIR
- ESFRI: European Strategy Forum on Research Infrastructures, a strategic instrument that was formed in 2002 at the behest of the European Council to develop the scientific integration of Europe and to strengthen its international outreach
- FAIR: The FAIR Data Principles propose that all scholarly output should be Findable, Accessible, Interoperable and Reusable [2]
- Galaxy: an open, Web-based platform for data-intensive biomedical research (https://usegalaxy.org/)
- GOBLET: Global Organisation for Bioinformatics Learning, Education and Training (http://www.mygoblet.org/), established in 2012 by an international group of societies and networks to offer an umbrella for bioinformatics, biocuration, biocomputing and computational biology learning, education and training
- GO FAIR: a proposal for the practical implementation of the European Open Science Cloud
- Health-RI: national infrastructure for personalized medicine and health research (https://www.health-ri.org)
- ISCB: International Society for Computational Biology (https://www.iscb.org/)
- ISMB: Intelligent Systems in Molecular Biology, the annual conference of the ISCB
- NBIC: Netherlands Bioinformatics Centre (https://www.nbic.nl/), one of the NGI-funded technology centres
- NCSB: Netherlands Consortium for Systems Biology, one of the NGI-funded technology centres
- NGI: Netherlands Genomics Initiative, an NWO-funded initiative with the aim to advance Dutch genomics research and to valorize the results, active in the period 2002–13
- NLeSC: Netherlands eScience Center (https://www.esciencecenter.nl/), the Dutch national hub for the development and application of domain overarching software and methods for the scientific community
- NWO: Netherlands Organization for Scientific Research (https://www.nwo.nl/en), the main governmental funding organization in the Netherlands
- PAC: Partner Advisory Committee of DTL, consisting of mandated representatives of the DTL Partners and advising the DTL Board
- RSG: Regional Student Group, a group of bioinformatics PhD students, which is part of the worldwide network of RSG’s coordinated by the Student Council of the International Society for Computational Biology (ISCB)
- SURF: the collaborative ICT organization for Dutch education and research (https://www.surf.nl/en/about-surf)
- SURFsara: one of SURF’s three operating companies. It creates a bridge between research and advanced ICT
- Software and Data Carpentry (SWC/DC, https://software-carpentry.org/, http://www.datacarpentry.org/): initiatives to teach researchers computing skills [13]
- Technology Hotels: expert groups that offer their high-end technologies and the associated expertise and infrastructure to researchers who do not have access to such facilities at their home institute
- TeSS: the online training portal of ELIXIR (https://tess.elixir-europe.org)
- YoungCB: Young Computational Biologists (http://www.youngcb.nl/): when BioSB came into existence, the RSG Netherlands broadened its scope and also embraced young researchers in Systems Biology, and changed its name into YoungCB
- ZonMw: the Netherlands Organisation for Health Research and Development (https://www.zonmw.nl/en/)

##

**Key Points**

- In the past 15 years, a strong bioinformatics community has been built in the Netherlands, combining bioinformatics research, support and training. This was facilitated by the collaborative Dutch culture in a relatively well-connected and small country.
- We learned that bioinformatics solutions should not be exclusively provided as lists of databases and tools; providing expert support is essential to find the right way forward.
- A central-distributed model was found to be successful for complex bioinformatics engineering projects. In this model, programmers are embedded in the life science labs, managed centrally and convening frequently to align the project developments and to learn from one another.
- We found that developing courses with involvement of staff from multiple institutions is beneficial for the quality and positioning of courses. This fosters ties among the Dutch bioinformatics and life science communities as a desirable side effect.
- New sustainability models including membership fees and pay-for-use can be successful if complemented by modest public funding for core infrastructural elements.
